# Cardiac involvement in established idiopathic inflammatory myopathy assessed by cardiac magnetic resonance mapping

**DOI:** 10.1007/s10067-025-07530-9

**Published:** 2025-06-12

**Authors:** Kateryna Yurchenko, Pil Højgaard, Redi Pecini, Sine S. Korsholm, Axel C. P. Diederichsen, Jesper Lindhardsen, Eva Søndergaard, Amalie D. Haue, Kasper Søltoft, Søren Jacobsen, Louise P. Diederichsen

**Affiliations:** 1 Center for Rheumatology and Spine Diseases, COPEACT, Copenhagen University Hospital, Blegdamsvej 9, 2100 Rigshospitalet, Copenhagen Denmark; 2 Department of Internal Medicine, Clinic of Rheumatology, Holbæk Hospital, Holbæk, Denmark; 3 Center for Rheumatology and Spine Diseases, Copenhagen University Hospital, Rigshospitalet, Frederiksberg, Denmark; 4 Department of Cardiology, Copenhagen University Hospital, Rigshospitalet, Copenhagen Denmark; 5 Department of Rheumatology, Odense University Hospital, Odense, Denmark; 6 Department of Cardiology, Odense University Hospital, Odense, Denmark

**Keywords:** Cardiac MRI, Established IIM, IBM, Myocardial involvement, Myositis, T1 mapping, T2 mapping

## Abstract

**Objectives:**

To investigate the prevalence of subclinical, myocardial involvement in patients with established idiopathic inflammatory myopathies (IIM) compared to healthy controls using T1 and T2 mapping by cardiac magnetic resonance imaging (CMRI).

**Method:**

Fifty-five patients with established, stable IIM without overt cardiac involvement were consecutively enrolled in this cross-sectional study. All patients completed questionnaires, underwent clinical examination, blood tests including antibody profiling, electrocardiography, and CMRI with T1 and T2 mapping. Concurrently, CMRI was conducted on 19 healthy controls. Abnormal T1 and T2 values were defined as values exceeding the 95th percentile in the control group. Potential associations between abnormal T1 and T2 values and various cardiac- and IIM-related outcomes were assessed in exploratory analyses.

**Results:**

Abnormal T1 values were observed in 9% of all IIM patients, displaying significantly higher T1 values compared to healthy controls (*p* = 0.02). T2 values were elevated in 18% of IIM patients, particularly among patients with non-inclusion body myositis (IBM) IIM compared to healthy controls (*p* = 0.03). No significant associations between T1 or T2 values and cardiac or disease-related measures were found in the present cohort of IIM patients.

**Conclusions:**

Our study demonstrates that subclinical cardiac involvement may be present in established, stable IIM patients, with abnormal T1 and T2 mapping observed in up to 18% of the cohort. These findings underscore the importance of ongoing cardiac monitoring, even during stable phases of the disease. However, prospective studies are needed to determine the prognostic value of T1 and T2 mapping in this disease entity.

**Table:** 

**Key Points** • *T1 and T2 mapping on cardiac MRI identify subclinical myocardial involvement in patients with IIM*. • *Subclinical myocardial involvement is present in up to 18% of patients with established, stable IIM*. • *Cardiac T1 and T2 abnormalities are rare in patients with IBM*.

**Supplementary Information:**

The online version contains supplementary material available at 10.1007/s10067-025-07530-9.

## Introduction

Idiopathic inflammatory myopathies (IIMs), collectively known as myositis, are rare autoimmune diseases primarily affecting skeletal muscles. The various subtypes of IIMs include dermatomyositis (DM), polymyositis (PM), immune-mediated necrotizing myopathy (IMNM), antisynthethase syndrome (ASyS), clinical amyopathic dermatomyositis (CADM), overlap myositis (OM), and inclusion body myositis (IBM) [1]. These subtypes exhibit variations in clinical manifestations, autoantibody profiles, prognoses, and treatment responses, with IBM having distinct demographic and clinical features [1, 2].

Chronic muscle inflammation in IIMs leads to progressive weakness, while persistent systemic inflammation can impact organs like the skin, joints, lungs, gastrointestinal tract, and the heart.

Cardiac involvement, alongside a higher prevalence of malignancies, is the main cause of mortality in IIM patients [3, 4]. It affects up to 70% of IIM patients and is a significant cause of morbidity and mortality within the first year’s post-diagnosis [5]. Unfortunately, cardiac symptoms often go unnoticed, leading to delayed diagnosis [5]. Hence, subclinical myocardial inflammation appears to play a central role, contributing to structural changes, myocardial dysfunction, and conduction disturbances [6].

Cardiac magnetic resonance imaging (CMRI) is a sensitive, noninvasive tool for visualizing cardiac function and structural characteristics [7]. While traditional late gadolinium enhancement (LGE) has limitations regarding detecting diffuse cardiac fibrosis, T1 and T2 mapping within CMRI provide enhanced sensitivity for detecting early and diffuse myocardial fibrosis and inflammation, particularly relevant in IIM [8, 9]. Moreover, LGE requires administration of gadolinium-based agents limiting its use in patients with renal dysfunction.

T1 and T2 mapping have been investigated in former studies on IIM patients [10–15]. These studies predominantly focused on individuals with relatively newly diagnosed IIM and reported significantly elevated T1 and T2 values compared to healthy controls. Given these findings and considering the well-established increased cardiovascular (CV) morbimortality in IIM, we hypothesized that even patients with established and stable disease might have subclinical myocardial involvement detectable through T1 and T2 mapping.

In the present study, we aimed to investigate potential subclinical cardiac involvement in established, stable IIM patients, and to compare these findings across IIM subtypes and to healthy controls using T1 and T2 mapping by CMRI. Additionally, we aimed to explore potential correlations between abnormal T1 and T2 findings and other cardiac- and IIM-related outcomes.

## Methods

### Design and population

The study was designed as an observational, exploratory cross-sectional study. Patients with stable IIM were consecutively enrolled from routine care at Department of Rheumatology at Odense University Hospital, Denmark from January 2018. Patients were included in the present study if they were ≥ 18 years of age and fulfilled the EULAR/ACR Classification Criteria for Idiopathic Inflammatory Myopathies [16, 17], IBM according to the European Neuromuscular Centre (ENMC) 2011 research diagnostic criteria [18], or ASyS (ICD-10 code M35.8) according to criteria by Solomon et al. [19]. IMNM was defined histopathologically by prominent myofiber necrosis and regeneration with minimal or no lymphocytic inflammation by an experienced neuropathologist.

Exclusion criteria were other co-existing rheumatic inflammatory diseases except Sjogren’s Syndrome, contraindications for CMRI scan, or patients with any known cardiac diseases (including but not limited to previously diagnosed heart failure, coronary artery disease, cardiomyopathy, congenital heart disease, and valvular heart disease).

Data on CMRI from healthy controls retrieved from the same MR scanner was obtained from hospital personnel and their relatives.

### Ethics

The study complies with the Declaration of Helsinki and was approved by the locally appointed Ethics Committee. All included patients gave informed, written consent.

### Cardiac- and IIM-related measures

**Fig. 1:**
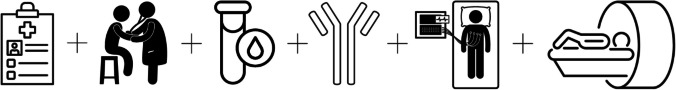
Illustration of the baseline assessment of all participants including completing the questionnaires, clinical examination, blood samples with autoantibody testing, electrocardiography, and cardiac magnetic resonance imaging (CMRI)

All participants underwent the various assessments at the day of the study visit (Fig. 1).

Patient characteristics including demographics, IIM subtype, time of diagnosis, and other disease-related features were obtained from the medical records. Information on comorbidities, medications, family medical history, heart symptoms, such as dyspnea, chest pain, palpitations, and smoking habits was collected through a questionnaire. Basic cardiac measures included height, weight, body mass index (BMI), systolic (S-BP), and diastolic (D-BP) blood pressure and 12-lead electrocardiography (ECG) at the clinical visit. ECG results were analysed according to standard criteria [20–22]. The corrected QT (QTc) interval was calculated using Bazett’s formula, i.e., QT duration divided by square root of RR interval. Prolonged QTc was defined as QTc duration ≥ 450 ms in any leads of V2, V3, or aVR.

Disease activity in IIM patients was assessed according to ACR/EULAR response Criteria for IIM including (1) patient-reported physical function by the Health Assessment Questionnaire (HAQ-DI), (2) Manual Muscle Test 8 (MMT8), which assesses the strength of 8 muscle groups in the body, (3) physician- and patient global disease activity/damage scores were obtained using a visual analogue scale (VAS) from 0 to 10 cm. VAS physician reflects the level of IIM disease burden based on the subject’s physical appearance, medical history, physical examination findings, laboratory results, and ongoing medical therapy, whereas VAS patient is a score of the overall impact of IIM symptoms from muscles, skin, joints, and internal organs [23–25].

### Laboratory testing

Blood samples analysis included cardiac markers (troponin T (TnT) and troponin I (TnI), C-reactive protein (CRP), creatine kinase (CK), and an autoantibody profile including myositis-specific autoantibodies (MSAs): anti-Jo-1, anti-PL-7, anti-PL-12, anti-EJ/OJ/KS, anti-SRP, anti-HMGCR, anti-MDA5, anti-TIF1γ, anti-NPX2, anti-SAE1, anti-Mi-2, anti-cN1A, and myositis associated autoantibodies (MAAs): anti-PM/Scl75, anti-PM/Scl100, anti-Ro52, anti-Ku, and anti-U1RNP.

### CMRI acquisition and analysis

CMRI was performed on a Philips Ingenia 1.5 T scanner with Omega HP gradient system (Philips Electronics, Koninklijke, Netherlands). Cine short-axis images enclosing the ventricles, as well as single cine views in four, three, and two chamber views were acquired with bSSFP sequences using slices of 8-mm thickness. A 32 channel Stream Torso coil was used during image acquisition. To null the myocardium, optimal inversion time (TI) was determined using a Look-locker sequence with multiple images with varying TI. The images were again acquired using 8-mm slices. The four, three, and two chamber views were acquired as a single slice while the short axis view as consecutive slices covering the whole ventricles. The T1 mapping sequences were acquired as a single midventricular slice in the short axis view. The modified Look-Locker inversion recovery sequence with a 5(3)3 sampling was used for the T1 images. For the T2 mapping sequence, eight images with different echo times were acquired following a T2 magnetization preparation.

Images were analysed in a blinded fashion by an experienced examiner with a CVI42® Software, version 5.13.2 (Circle Cardiovascular Imaging Inc., Calgary, Alberta, Canada). Both the functional assessments (volumes, myocardial mass) and the T1 and T2 values were measured automatically with the help of AI-based software and the contours were checked for accuracy afterwards. To ensure that the T1 values represented myocardial tissue exclusively, a 20% offset limit was used for both the endocardial and epicardial border.

Abnormal T1 and T2 values were predefined as those exceeding the 95 th percentile of the values observed in the control group corresponding to > 1055 ms for T1 and > 56 ms for T2.

### Statistical analysis

Demographics, CV comorbidities, medications, and clinical, laboratory, and imaging measures were reported as percentages for binary variables and as means ± SD or medians (IQR) for continuous variables, based on normality of distribution.

Comparison of means between groups was performed by independent samples T-tests after ensuring that (1) the scores were normally distributed in each group and (2) the assumption of homogeneity of variance was fulfilled by applying Levene’s test of variance.

In case of non-normally distributed, continuous data, comparisons between groups were made by the nonparametric Kruskal–Wallis test. Categorical data were compared using Pearson’s *χ*^2^ test. *P* < 0.05 was considered significant, and all statistical tests were two-tailed tests.

We also explored the relationship between abnormal T1 and T2 values and several parameters of interest, including demographic characteristics, disease-specific measures, cardiovascular risk factors, measures of TnT/TnI, ECG parameters (QTc and QRS duration), and CMRI-derived structural and functional heart parameters with use of Fisher’s exact test (categorical variables) or the Wilcoxon rank sum test (continuous variables), as appropriate.

Statistical analyses were performed using SPSS v. 28 (IBM software) and R version 4.2.0.

## Results

**Fig. 2:**
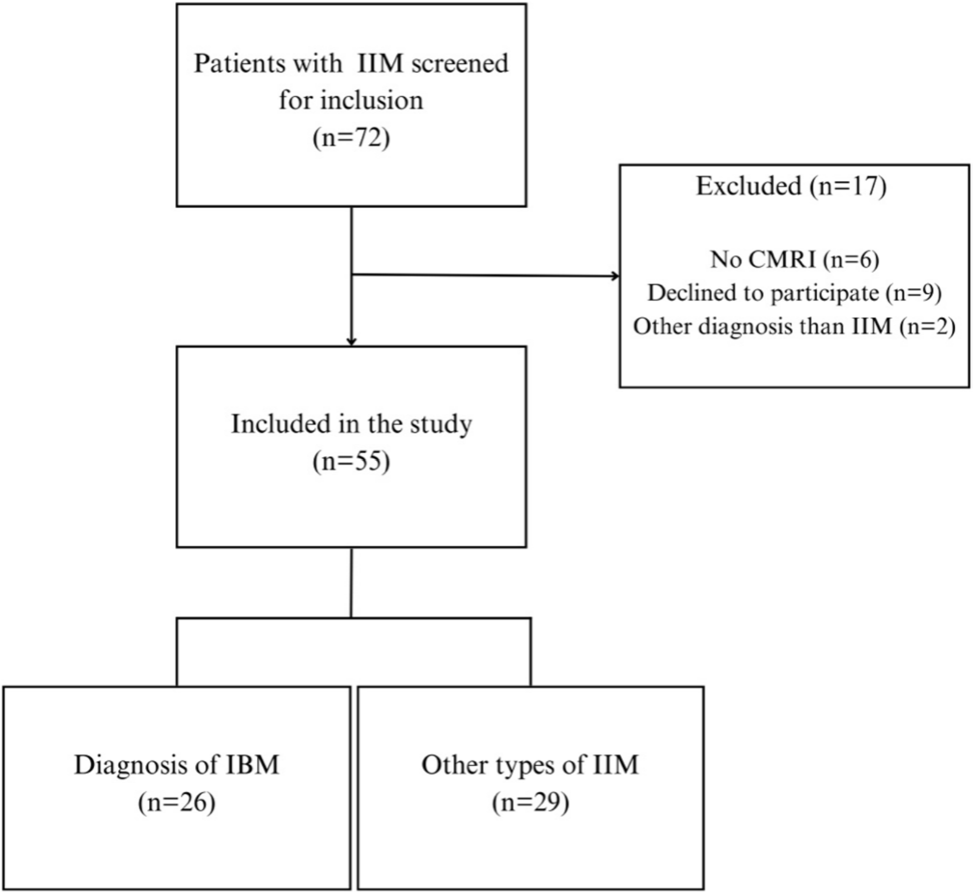
Flowchart of the inclusion procedure in this study. IIM, idiopathic inflammatory myopathy; IBM, inclusion body myositis; CMRI, cardiac magnetic resonance imaging

During the enrolment period, a total of 72 patients were screened for inclusion. Of these, two patients did not fulfil the criteria for an IIM diagnosis, and nine patients declined to participate. Hence, 61 patients were included in the study. Six of these patients did not undergo CMRI due to technical or practical issues, leaving 55 patients with established and stabile IIM available for study analyses (Fig. 2). The demographic characteristics of the patient population along with IIM subsets, clinical characteristics, and disease activity measurements are reported in Table 1.

**Table 1:** Demographic and clinical characteristics of patients with idiopathic inflammatory myopathy (IIM)

	All IIM^*^ (*n* = 55)	**Non-IBM IIM (*n* = 29)**	**IBM (*n* = 26)**	***P*-value**^**a**^
**Characteristics**				
Age, years	63.7 ± 12.5	57.9 ± 13.3	70.2 ± 7.5	** < 0.001**
Female, *n* (%)	34 (62)	22 (76)	12 (46)	**0.02**
Caucasian, *n* (%)	54 (98)	28 (97)	26 (100)	
IIM Disease duration, years	5.0 ± 4.9	4.0 ± 3.8	6.2 ± 5.7	0.1
IIM Symptom duration, years	8.0 ± 5.9	5.7 ± 4.2	10.5 ± 6.5	**0.002**
**IIM subsets, *n* (%)**				
Polymyositis	5 (9)	5 (17)		
Amyopathic dermatomyositis	10 (18)	10 (35)		
Antisynthetase	7 (13)	7 (24)		
Dermatomyositis	5 (9)	5 (17)		
Immune-mediated necrotizing myopathy	2 (4)	2 (7)		
**Clinical characteristics**				
Raynaud, *n* (%)	8 (15)	6 (21)	2 (8)	0.12
Rash, *n* (%)	19 (35)	17 (59)	2 (8)	** < 0.001**
Arthritis, *n* (%)	11 (20)	11 (38)	0 (0)	** < 0.001**
Interstitial lung disease, *n* (%)	10 (18)	9 (31)	1 (4)	**0.01**
Dysphagia, *n* (%)	30 (55)	15 (52)	15 (58)	1.0
**Disease activity measures**				
Creatinekinase, u/L ^, (*n* = 53)	143 (151)	121 (90)	198.5 (233)	0.13
Increased, *n* (%)	9 (17)	1 (3)	8 (31)	**0.009**
Manual muscle test, MMT8 (0–80)	76 (9)	78 (3)	68 (15)	** < 0.001**
Health assessment questionnaire, HAQ-DI (0–3)	0.625 (1.25)	0.250 (0.750)	1.125 (1.813)	**0.005**
Physician global activity, VAS 0–100 (*n* = 52)	3 (8)	5 (10)	2.5 (4)	**0.01**
Patient global activity, VAS 0–100, (*n* = 52)	3 (15)	10 (20)	0 (5)	**0.03**
Global extramuscular activity, VAS 0–100 (*n* = 52)	0 (5)	5 (10)	0 (4)	0.08
**Disease damage measures**				
Physician global damage, VAS 0–100 (*n* = 51)	25 (35)	20 (18)	40 (35)	** < 0.001**
Patient global damage, VAS 0–100 (*n* = 51)	30 (38)	30 (40)	39.5 (26)	0.13
**Myositis autoantibodies positive patients, *n* (%)**	**40 (73)**	**24 (83)**	**16 (61.5)**	
**Myositis-specific autoantibodies positive, *n* (%)**	**37 (67)**	**23 (79)**	**14 (53.8)**	
Anti-Jo1	5 (9)	5 (17)	0 (0)	
Anti-PL7	2 (4)	2 (7)	0 (0)	
Anti-PL12	0 (0)	0 (0)	0 (0)	
Anti-SRP	4 (7)	3 (10)	1 (4)	
Anti-HMGCR	2 (4)	2 (7)	0 (0)	
Anti-MDA5	4 (7)	4 (14)	0 (0)	
Anti-TIF-y	4 (7)	3 (10)	1 (4)	
Anti-SAE1	2 (4)	2 (7)	0 (0)	
Anti-NXP2	2 (4)	2 (7)	0 (0)	
Anti-cN1A	16 (29)	3 (10)	13 (50)	
**Myositis-assotiated autoantibodies positive, *n* (%)**	**18 (33)**	**12 (41.4)**	**6 (23.1)**	
Anti-Ro52	18 (33)	12 (41)	6 (23)	
Anti-PM/Scl75 and 100	1 (2)	1 (3)	0 (0)	
Anti-Ku	0 (0)	0 (0)	0 (0)	
**Immunosupressives, *n* (%)**				
Prednisolone	19 (34)	15 (52)	4 (15)	**0.005**
sDMARD^**^	32 (58)	25 (86)	7 (27)	** < 0.001**
bDMARD^***^	1 (2)	1 (3)	0 (0)	0.34
IVIG	2 (4)	2 (7)	0 (0)	0.17

^*^IIM, idiopathic inflammatory myopathy including dermatomyositis, polymyositis, immune-mediated necrotizing myositis, antisynthetase syndrome; *IBM*, inclusion body myositis

^Reference interval: 40–280 U/L

^**^sDMARD, synthetic disease-modifying antirheumatic drugs

^***^bDMARD, biological disease-modifying antirheumatic drugs

^a^*p*-value for difference between patients with IIM other than IBM vs. patients with IBM. Values are *n* (%) for categorical data and mean (± SD) or median (IQR) for continuous data depending on data distribution. Two-sided *p*-values are shown for categorical data compared by chi-square tests; means were compared by independent samples *T*-tests, medians by nonparametric independent samples median test

The healthy control group consisted of 19 participants with a mean age of 48 (± 17) and 8 (42%) were males.

### Cardiovascular outcomes

Table 2 summarizes the CV characteristics according to IIM subgroup. Hypercholesterolemia and hypertension were common comorbidities, the latter being significantly more prevalent in IBM versus patients with non-IBM IIM. The blood test results showed that 94% of all IIM patients had TnI levels within the normal range (< 5 ng/L), 3 patients (6%) had levels between 5 and 10 ng/L, and none had TnI levels above 10 ng/L. Elevated TnT levels were more commonly observed in patients with IBM compared to non-IBM IIM (45% vs. 18%). The most common ECG abnormality was prolonged QTc (> 450 ms) which was found in 9 (17%) of all IIM patients.

**Table 2:** Cardiovascular characteristics and risk factors in patients with idiopathic inflammatory myopathy (IIM)

	All IIM (*n* = 55)	Non-IBM IIM (*n* = 29)	IBM (*n* = 26)	*p*-value^a^
**CV risk factors**				
Smoking status, *n* (%)				
Current	5 (9)	2 (7)	3 (12)	0.55
Former	19 (34)	10 (35)	9 (35)	0.99
Never	31 (56)	17 (59)	14 (54)	0.72
BMI (kg/m^2^), mean	28.0 ± 6.6	30.3 ± 7.9	25.6 ± 4.1	**0.009**
BMI > 29.9, *n* (%)	14 (25)	11 (38)	3 (12)	**0.02**
Family history of CVD, *n* (%)^*^	22 (40)	10 (35)	12 (46)	0.38
**Current comorbidities, *n* (%)**				
Diabetes^**^ HbA1c > 48 mmol/mol	8 (14) 3 (5)	3 (10) 0 (0)	5 (19) 3 (12)	0.35 NA
Hypercholesterolemia^***^ Plasma cholesterol > 5.0 mmol/L	36 (65) 30 (55)	19 (66) 19 (66)	17 (65) 11 (42)	0.99 NA
Hypertension^****^ Systolic BP > 140 mm-Hg	37 (67) 22 (40)	16 (55) 12 (41)	21 (81) 10 (34)	**0.04** NA
**Cardiac symptoms *n* (%)**				
Dyspnea	19 (34)	13 (45)	6 (23)	0.09
Palpitations (*n* = 52)	17 (32)	9 (31)	8 (31)	0.77
Syncope (*n* = 50)	3 (6)	2 (7)	1 (4)	0.55
**Laboratory results**				
Troponin I (*n* = 50)				
< 5 ng/L, *n* (%)	47 (94)	27	20	0.42
> 5 ng/L, n (%)	3 (6)	1 (3)	2 (8)	0.42
< 10 ng/L, n (%)	0 (0)	0 (0)	0 (0)	
Troponin T ng/L, median (*n* = 51)	24 (43)	11 (16.3)	43 (63)	** < 0.001**
> 14 ng/L, *n* (%)	32 (63)	9 (31)	23 (89)	** < 0.001**
**ECG results (*n* = 53)**				
Atrial fibrillation/flutter, *n* (%)	1 (2)	0 (0)	1 (4)	0.29
PQ-duration (ms), mean	163.8 ± 29.9	151.2 ± 20.3	178.5 ± 32.8	** < 0.001**
> 220 ms, *n* (%)	2 (4)	0 (0)	2 (8)	0.12
QRS-duration (ms), mean	92.5 ± 15.9	90.5 ± 11.8	94.8 ± 19.6	0.33
> 120 ms, *n* (%)	2 (4)	0 (0)	2 (8)	0.13
QTc-duration (ms), mean	428.4 ± 23.4	424.3 ± 22.7	433.0 ± 23.6	0.18
> 450 ms, *n* (%)	9 (17)	3 (10)	6 (23)	0.20

*IIM*, idiopathic inflammatory myopathy including dermatomyositis, polymyositis, immune-mediated necrotizing myositis, antisynthetase syndrome, amyopathic dermatomyositis; *IBM*, inclusion body myositis; *BMI*, body mass index; *CV*, cardiovascular; *BP*, blood pressure

^*^Family history of CV disease including premature heart disease in first-degree relatives; in men < 55 years, and in women age < 65 years

^**^Defined as HbA1c > 48 mmol/mol or use of antidiabetics

^***^Defined as plasma cholesterol > 5.0 mmol/L or use of statins

^****^Defined as systolic BP > 140 mm-Hg or use of antihypertensiva

^a^*p*-value for difference between patients with IIM other than IBM vs. patients with IBM

### CMRI

Table 3 provides a summary of the findings from CMRI measures in patients with IIM compared between subgroups and healthy controls.

**Table 3:** CMRI findings in patients with idiopathic inflammatory myopathy (IIM) compared to subgroups and healthy controls

	Non-IBM IIM (*n* = 29)	IBM (*n* = 26)	*P*-value^a^	All IIM (*n* = 55)	Healthy controls (*n* = 19)	*P*-value^b^
**Morphological and functional parameters**						
LVEDV, mL/m^2^	139.1 ± 39.9	124.4 ± 24.8	0.11	132.2 ± 34.1	166.0 ± 45.8	**0.001**
LVESV, mL/m^2^	46.7 ± 17.7	41.8 ± 13.9	0.27	44.4 ± 16.0	58.8 ± 18.9	**0.002**
LVEF, %	67.0 ± 5.6	66.7 ± 6.5	0.87	66.8 ± 6.0	64.8 ± 4.6	0.19
RVEDV, mL/m^2^	156.2 ± 53.2	131.3 ± 31.8	**0.04**	144.5 ± 45.8	185.2 ± 54.3	**0.002**
RVESV, mL/m^2^	67.4 ± 28.2	56.0 ± 19.6	0.09	62.0 ± 25.0	84.9 ± 28.7	**0.001**
RVEF, %	57.3 ± 5.5	57.9 ± 7.3	0.73	57.6 ± 6.4	54.5 ± 5.7	0.06
AoSV, mL/m^2^	79.6 ± 21.4	68.1 ± 16.1	**0.03**	74.1 ± 19.8	94.37 ± 20.8	** < 0.001**
PulmSV, mL/m^2^	81.3 ± 20.7	68.0 ± 16.0	**0.01**	75.0 ± 19.6	95.4 ± 24.5	** < 0.001**
**Mapping parameters**						
T1 native, msec	1008.9 ± 36.9	999.5 ± 39.5	0.37	1004.5 ± 38.0	980.1 ± 38.1	**0.02**
T2 msec*	54.5 (7)	52.5 (4)	**0.03**	53.5 (6)	51.0 (4)	0.10

*LVEDV*, left ventricle end-diastolic volume; *LVESV*, Left ventricle end-systolic volume; LVEF, Left ventricle ejection fraction; *RVEDV*, right ventricle end-diastolic volume; *RVESV*, right ventricle end-systolic volume; RVEF, right ventricle ejection fraction; *AoSV*, aortic stroke volume; *PulmSV*, pulmonary stroke volume

^a^*p*-value for difference between patients with IIM other than IBM vs. patients with IBM

^b^*p*-value for difference between patients with All patients with IIM and healthy controls. Values are means (± SD) or median (IQR). Means were compared by independent samples T-tests, medians by nonparametric independent samples median test

^*^(*n* = 48)

Notably, all IIM patients displayed overall significantly poorer morphological heart parameters when compared to the healthy controls but had preserved ejection fractions (EFs). In comparison to non-IBM IIMs, IBM patients exhibited significantly lower right ventricular diastolic volume (RVEDV), aortic, and pulmonary systolic volumes (AoSV, PulmSV).

**Fig. 3:**
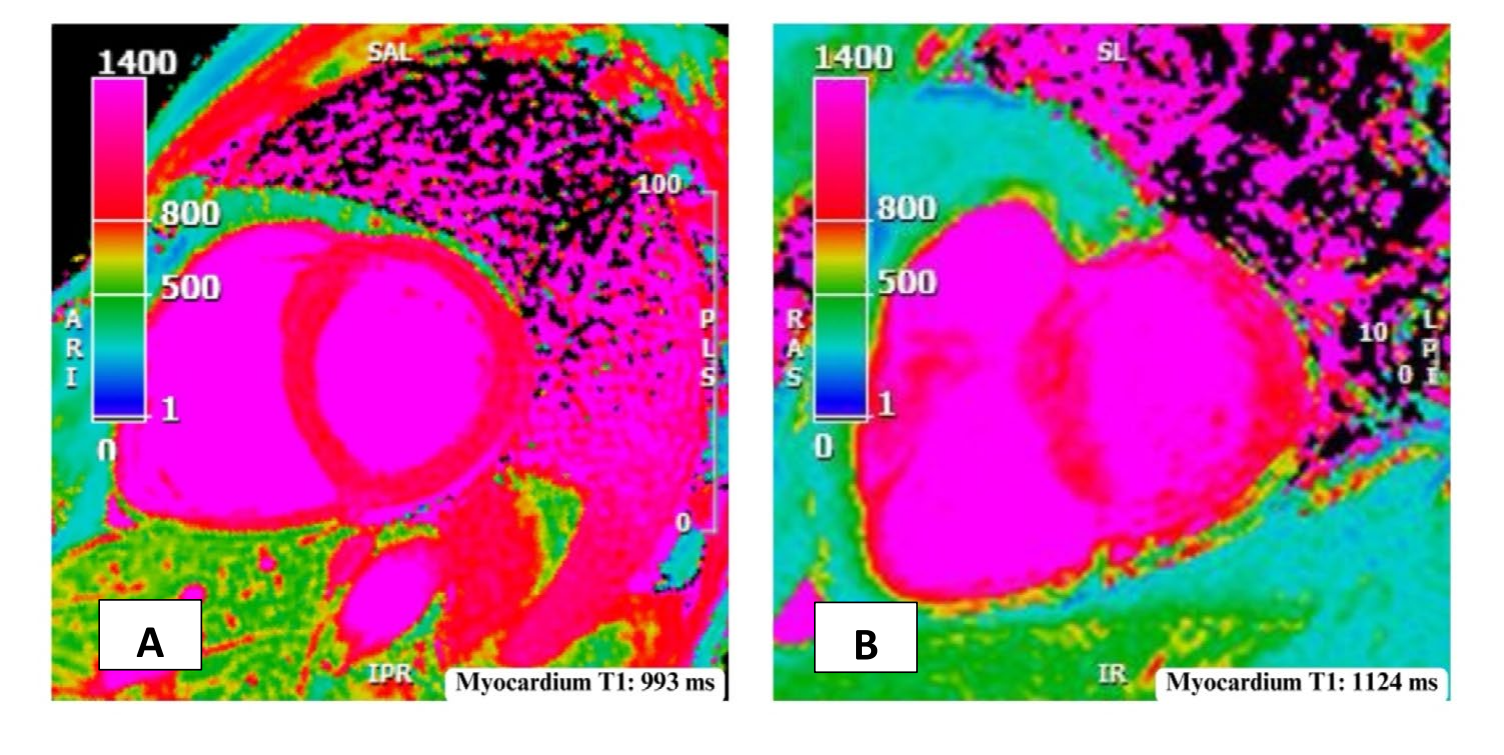
Illustration of native T1 assessment in **A** a healthy control (993 ms) and **B** a patient with idiopathic inflammatory myopathy (1124 ms)

Significantly higher native T1 values were observed among patients with IIM compared to healthy controls, but no significant difference was found in native T1 values between the IIM subgroups. Five out of 55 IIM patients (9%) exhibited abnormal T1 values according to the predefined criteria, including one patient with IBM and four patients with non-IBM IIM subtypes. Figure 3 provides an illustration of native T1 mapping in a healthy control and a patient with non-IBM IIM.

No significant difference in T2 values was observed between the overall IIM cohort and healthy controls. However, T2 values were significantly higher in non-IBM IIM patients compared to those with IBM. Further analysis revealed a significant difference in T2 values between non-IBM IIM patients and healthy controls (*p* = 0.03), whereas no significant difference was found between patients with IBM and healthy controls (*p* = 0.648), data not shown elsewhere. Ten (18%) non-IBM IIM patients exhibited abnormal T2 values according to the predefined criteria.

Three patients exhibited both abnormal T1 and T2 values. All three were females, aged 29, 46, and 63 at the time of CMRI and diagnosed with ASyS, PM, and DM, respectively. No pattern was identified regarding the autoantibody profile and abnormal T1 and T2 mapping values.

### Cardiac mapping parameters in association to cardiac and disease-related parameters in IIM patients

Exploratory analyses revealed an association between abnormal T1 values and increasing BMI in IIM patients, while no other significant positive correlations with the cardiac- or disease-related characteristics were identified (Supplementary Table 1). On the contrary, a negative association was identified between abnormal T1 values and cardiovascular risk factors as increasing systolic blood pressure, hypertension, and hypercholesterolemia.

There were no significant associations between abnormal T2 values and cardiac or disease-related parameters in patients with non-IBM IIM, except a negative correlation between abnormal T2 values and hypercholesterolemia (Supplementary Table 2).

## Discussion

This study assessed myocardial involvement in established and stable patients with various subsets of IIM using CMRI with T1 and T2 mapping, comparing them to healthy controls. We also examined correlations between abnormal T1/T2 values and cardiac and disease-related outcomes in IIM patients.

Our investigation revealed elevated native T1 values in patients with IIM in comparison to healthy controls. Using predefined criteria based on our healthy controls, five of the 55 IIM patients (9%) showed abnormal T1 values. According to Ferriera et al., T1 alone might not suffice to distinguish between acute and chronic myocardial diseases [26]. However, they acknowledge its sensitivity as a marker for diseased myocardium by observing a correlation between tissue alterations in the heart, such as diffuse inflammation and fibrosis, and elevated T1 mapping values [26]. This observation indicates a higher prevalence of inflammation and fibrosis within our IIM patient cohort, aligning with our hypothesis that even patients with established and stable disease might have subclinical myocardial involvement detectable by T1 mapping.

Our findings are in accordance with previous case–control studies on patients with IIM showing increased native T1 value in patients compared to controls [10–15]. However, the proportion of IIM patients exhibiting abnormal T1 values (40–90.9%) in these studies significantly exceeds our population (9%). This discrepancy can in part be explained by differences in study design and study population. Three of the aforementioned studies are retrospective, which inherently limits the control of population sampling, potentially impacting the accuracy of the findings [12, 13, 15]. Additionally, five of six studies involve Asian populations, which might have an impact on the results [10, 11, 13–15].

A key distinction between our study population and those in previous studies is the disease duration. In prior studies, mean disease duration was less than 15 months versus mean disease duration of 5 years in our cohort of established and stable patients. Additionally, most of the prior studies reported that 87–100% of their patients were receiving steroid treatment at the time of CMRI, compared to only 34% in our cohort. Since these studies did not report disease activity measures according to ACR/EULAR, we can only infer from the available findings that their patient cohorts may have had more active disease [24]. This potential difference in disease activity could explain the higher proportion of abnormal T1 values in those studies.

Only one previous study has used T1 and T2 mapping techniques in established and stable IIM patients without clinical cardiac involvement [27]. In this cohort of 22 stable IIM patients, increased T1 values were identified in 18.2% (4/22). However, the study focused on a single IIM subtype—antisynthetase syndrome—which complicates direct comparison.

In the present study, comparation of T2 values between all IIM patients and healthy controls did not reach significance. However, T2 values were significantly higher in non-IBM IIM patients compared to both IBM patients and healthy controls. T2 mapping is an advanced imaging technique recognized for its reliability in detecting myocardial oedema, a hallmark of inflammation, suggesting that T2 mapping is more specific for identifying acute myocardial inflammation compared to T1 mapping [26]. Our findings point to a significant difference in myocardial oedema between non-IBM IIM and IBM patients in our cohort, potentially related to ongoing subclinical cardiac inflammation in the non-IBM subgroup. This result aligns with the understanding that IBM mainly affects skeletal muscles and does not typically increase the risk of heart problems [28]. This is further supported by our results, which specifically show no significant difference in T2 values between patients with IBM and healthy controls.

Among prior CMRI studies, four Asian case–control studies reported significantly prolonged T2 values in newly diagnosed IIM patients compared to healthy controls [11–13, 15], a result we were unable to replicate in the overall cohort of IIM patients. Similar to T1 mapping, the proportion of abnormal T2 values in our study was lower (18%) compared to the range reported in these studies (40–73.7%). Notably, none of the patients with IBM in our cohort exhibited abnormal T2 values, which may partly explain the differences, as those studies didn’t include patients with IBM. In the non-IBM subgroup, abnormal T2 values were observed in 34.5% (10/29) of patients. The study on stable ASyS patients reported elevated T2 values in 13.6% (3/22) of patients [27]. The presence of abnormal T2 values in our stable non-IBM IIM cohort suggests that some patients may experience ongoing cardiac inflammation despite being in a stable disease phase. This finding may underscore the importance of continued cardiac monitoring in non-IBM IIM patients.

Our exploratory association analyses did not identify any positive correlations between T1 and T2 values and cardiological or disease-related parameters, except for a significant association between abnormal T1 values and increasing BMI in IIM. Notably, there was an inverse association between abnormal T values and presence of traditional cardiovascular risk factors as hypertension and hypercholesterolemia in IIM, and between abnormal T2 values and hypercholesterolemia in non-IBM IIM. Furthermore, additional cardiac risk factors as age, gender, and smoking did not correlate with abnormal T1 and T2 values. Taken together, these findings indicate that traditional cardiovascular risk factors are unlikely to account for the T-mapping abnormalities. To our knowledge, only one previous study has conducted association analyses between IIM-related parameters and cardiac native T1 values [10]. This study identified a correlation between elevated NT-proBNP levels and T1 values in patients with PM and DM. However, TnT and CK levels as well as disease duration did not show any significant correlation with T1 values. Nonetheless, the absence of ACR/EULAR disease activity measures for IIM in their study limits the comparability of their results with ours.

The primary strength of this study is its novelty in reporting cardiac T1 and T2 mapping values for a cohort of established and stable patients across various IIM subtypes. Furthermore, the cross-sectional design with consecutively enrolled patients provides a methodological advantage over the retrospective designs commonly used in prior CMRI studies on patients with IIM. Finally, our study includes the extensive profiling of IIM-specific core set measures, including detailed assessments of disease activity and damage status, a complete antibody profile, and comprehensive cardiac parameters.

Some study limitations should be noted. This is a single-center study, involving a relatively small number of patients with various subtypes of IIM. Given that IIM is a rare disease, and the prevalence of cardiac involvement among IIM patients ranges widely from 6 to 75%, there is a considerable risk of type 2 error in the current, exploratory study due to lack of power. Still, this study includes the largest IIM cohort to date on CMRI. Despite comprehensive evaluation, we did not identify any correlations between IIM-related outcomes and T1 or T2 mapping values. However, the limited number of IIM patients with abnormal T1 and T2 values in these correlation analyses does not allow any firm conclusion to be drawn regarding potential biomarkers of cardiac involvement.

Another limitation in our study is the absence of measures of LGE. LGE is a traditional method for detecting heart fibrosis and it could potentially complement the findings from the more sensitive CMRI mapping techniques regarding cardiac involvement. However, we focused specifically on subclinical cardiac involvement assessed by T1 and T2 mapping, as only patients without clinical heart involvement were included.

Finally, our healthy controls were not sex and age matched with the IIM patients. However, evidence suggests that age has a limited effect on cardiac T1 and T2 mapping results, whereas sex appears to play a role, in particular significant prolongation of T1 relaxation time in females [29, 30].

## Conclusion

Our study demonstrates that 9% of all patients with stable IIM and normal EF exhibited abnormal T1 values, and 18% of non-IBM IIM patients showed abnormal T2 values, based on thresholds established from healthy controls. These results align with our hypothesis that even in stable phases of disease, patients with IIM may still experience subclinical cardiac involvement, including diffuse fibrosis and ongoing inflammation. Despite the comprehensive assessment of our patients, we did not identify any disease-related or cardiac biomarkers associated with subclinical heart involvement in this population. Our results highlight the importance of continued cardiac monitoring, even in the absence of active disease signs. However, the potential treatment of subclinical cardiac involvement remains an unknown territory. Additionally, further prospective studies are needed to clarify the prognostic value of CMRI for cardiac outcomes in patients with IIM.

## Supplementary Information

Below is the link to the electronic supplementary material.

Supplementary file1 (DOCX 24 KB)

## Data Availability

The data that supports the findings of this study is available on request from the corresponding author. The data are not publicly available due to patient privacy.
