# Supplementary material for: Cardiac involvement in established idiopathic inflammatory myopathy assessed by cardiac magnetic resonance mapping
Source: Clin Rheumatol. 2025 Jun 12;44(7):2941–50. doi: 10.1007/s10067-025-07530-9 (PMC12234596; doi:10.1007/s10067-025-07530-9)
Supplement: Supplementary file 1 — Supplementary file1 (DOCX 24 KB) [file 10067_2025_7530_MOESM1_ESM.docx]

Supplementary material

**Supplementary Table 1.** Association between abnormal T1-mapping values (95th Percentile) and IIM-related and cardiac characteristics in all IIM patients.

|  | **Normal T1**, N = 50^1^ | **Abnormal T1**, N = 5^1^ | **p-value**^2^ | |
| --- | --- | --- | --- | --- |
| **Demographics** |  |  |  |  |
| Female | 30 (60%) | 4 (80%) | | 0.6 |
| Age | 68 (58, 72) | 63 (46, 67) | | 0.11 |
| **IIM-related measures** |  |  |  |  |
| Diagnosis of IBM | 22 (44%) | 1 (20%) | | 0.4 |
| Disease duration | 3.5 (1.7, 6.8) | 2.6 (2.2, 3.7) | | 0.6 |
| Symptom duration | 7.4 (4.0, 11.6) | 3.6 (3.3, 5.5) | | 0.2 |
| Global extramuscular activity, VAS 0-100 | 0.0 (0.0, 5.0) | 0.0 (0.0, 5.0) | | 0.9 |
| Patient global damage, VAS 0-100 | 30 (13, 50) | 35 (30, 50) | | 0.9 |
| Physician global damage, VAS 0-100 | 25 (10, 49) | 20 (10, 30) | | 0.4 |
| Patient global activity, VAS 0-100 | 3 (0, 15) | 0 (0, 10) | | 0.6 |
| Physician global activity, VAS 0-100 | 3.0 (0.0, 7.0) | 0.0 (0.0, 5.0) | | 0.6 |
| Manual muscle test, MMT8 (0-80) | 76 (67, 78) | 77 (75, 78) | | 0.3 |
| Health assessment questionnaire, HAQ-DI (0-3) | 0.56 (0.13, 1.31) | 0.63 (0.00, 1.13) | | 0.6 |
| Creatinekinase | 142 (101, 239) | 247 (56, 267) | | 0.9 |
| Creatinekinase, > 280 U/L | 8 (17%) | 1 (20%) | | 0.9 |
| CRP | 2.2 (1.2, 5.0) | 3.2 (1.2, 4.8) | | 0.9 |
| **Extramuscular disease activity** |  |  |  |  |
| Rash | 18 (36%) | 1 (20%) | | 0.6 |
| Raynaud phenomenon | 8 (17%) | 0 (0%) | | 0.9 |
| Arthritis | 10 (21%) | 1 (20%) | | 0.9 |
| Interstitial lung disease | 9 (18%) | 1 (20%) | | 0.9 |
| Dysphagia | 28 (58%) | 2 (50%) | | 0.9 |
| **CV risk factors** |  |  |  |  |
| Dyspnea | 18 (36%) | 1 (20%) | | 0.6 |
| Angina | 5 (11%) | 1 (20%) | | 0.5 |
| Palpitations | 16 (34%) | 1 (20%) | | 0.9 |
| Syncope | 3 (6.5%) | 0 (0%) | | 0.9 |
| Blood pressure (systolic) | 138 (131, 146) | 128 (116, 134) | | **0.035** |
| Blood pressure (diastolic) | 84 (77, 91) | 82 (77, 82) | | 0.3 |
| BMI | 26 (23, 29) | 35 (27, 43) | | **0.042** |
| Hypertension | 36 (72%) | 1 (20%) | | **0.035** |
| Diabetes | 8 (16%) | 0 (0%) | | 0.9 |
| Hypercholesterolemia | 35 (70%) | 1 (20%) | | **0.043** |
| HbA1c | 37.0 (33.0, 39.5) | 37.0 (34.0, 37.0) | | 0.7 |
| **Smoking status** |  |  | | 0.15 |
| Never | 30 (60%) | 1 (20%) | |  |
| Former | 16 (32%) | 3 (60%) | |  |
| Current | 4 (8.0%) | 1 (20%) | |  |
| TnI | 1.00 (1.00, 2.10) | 1.00 (1.00, 1.00) | | 0.11 |
| TnI, > 5 ng/L | 3 (6.7%) | 0 (0%) | | 0.9 |
| TnT | 24 (11, 51) | 10 (7, 29) | | 0.4 |
| TnT, >14 ng/L | 29 (63%) | 2 (40%) | | 0.4 |
| **ECG** |  |  |  |  |
| **Sinus rhythm** |  |  | | 0.9 |
| 0 | 1 (2.1%) | 0 (0%) | |  |
| 1 | 47 (98%) | 5 (100%) | |  |
| Heart Rate | 70 (60, 77) | 81 (62, 84) | | 0.5 |
| P-wave-duration | 80 (80, 100) | 80 (80, 110) | | 0.9 |
| PQ-duration | 160 (140, 178) | 164 (158, 172) | | 0.4 |
| QT-duration | 408 (382, 424) | 378 (348, 386) | | 0.15 |
| QTc (Fredericia) | 422 (408, 435) | 396 (389, 422) | | 0.088 |
| QTc (Bassets) | 434 (411, 443) | 417 (412, 428) | | 0.3 |
| QRS-duration | 92 (84, 98) | 90 (90, 98) | | 0.9 |
| QRS-duration, >120ms | 2 (4.1%) | 0 (0%) | | 0.9 |
| **CMRI** |  |  |  |  |
| LVEDV | 122 (106, 138) | 152 (152, 160) | | **0.023** |
| LVESV | 39 (34, 49) | 59 (49, 65) | | **0.012** |
| LVEF | 67.0 (64.0, 70.8) | 64.0 (63.0, 67.0) | | 0.2 |
| RVEDV | 133 (110, 154) | 171 (162, 178) | | 0.063 |
| RVESV | 54 (44, 69) | 70 (68, 80) | | 0.053 |
| RVEF | 58 (55, 61) | 57 (53, 58) | | 0.6 |
| AoSV | 69 (58, 83) | 85 (83, 97) | | 0.079 |
| PulmSV | 71 (59, 86) | 87 (81, 91) | | 0.13 |
| Native T1- mapping | 997 (978, 1,015) | 1,082 (1,058, 1,089) | | **<0.001** |
| T2 - mapping | 52 (51, 55) | 57 (56, 58) | | 0.11 |
| ^1^ n (%); Mean (±SD) or Median (IQR). ^2^ Fisher’s exact test; Wilcoxon rank sum test.  IIM=idiopathic inflammatory myopathy; IBM = inclusion body myositis; CV= cardiovascular; LVEDV=Left ventricle end-diastolic volume volume; LVESV=Left ventricle end-systolic volume; LVEF=Left ventricle ejection fraction; RVEDV= Right ventricle end-diastolic volume; RVESV=Right ventricle end-systolic volume; RVEF=Right ventricle ejection fraction; AoSV= Aortic stroke volume; PulmSV= Pulmonary stroke volume. | | | | |

**Supplementary Table 2.** Association between abnormal T2-mapping values (95th Percentile) and IIM-related and cardiac characteristics in non-IBM IIM patients.

|  | **Normal T2**, N = 21^1^ | **Abnormal T2**, N = 9^1^ | **p-value**^2^ |
| --- | --- | --- | --- |
| **Demographics** |  |  |  |
| Female | 16 (76%) | 7 (78%) | >0.9 |
| Age | 59 (52, 67) | 55 (46, 63) | 0.3 |
| **IIM-related measures** |  |  |  |
| Diagnosis of IBM | 0 (0%) | 0 (0%) | >0.9 |
| Disease duration | 3.50 (1.74, 5.54) | 2.15 (1.61, 5.01) | 0.5 |
| Symptom duration | 5.5 (2.6, 7.6) | 2.9 (1.9, 9.2) | 0.3 |
| Global extramuscular activity, VAS 0-100 | 5 (0, 10) | 0 (0, 5) | 0.5 |
| Patient global damage, VAS 0-100 | 33 (10, 45) | 30 (12, 40) | >0.9 |
| Physician global damage, VAS 0-100 | 20 (10, 28) | 20 (6, 30) | 0.8 |
| Patient global activity, VAS 0-100 | 9 (0, 23) | 10 (0, 12) | 0.5 |
| Physician global activity, VAS 0-100 | 5.0 (0.0, 10.0) | 0.0 (0.0, 5.0) | 0.3 |
| Manual muscle test, MMT8 (0-80) | 77.00 (76.00, 80.00) | 77.0 (76.00, 80.00) | 0.5 |
| Health assessment questionnaire, HAQ-DI (0-3) | 0.25 (0.13, 0.63) | 0.25 (0.13, 0.63) | >0.9 |
| Creatinekinase | 133 (105, 174) | 75 (56, 111) | 0.077 |
| Creatinekinase, > 280 U/L | 2 (11%) | 0 (0%) | >0.9 |
| CRP | 2.2 (1.2, 5.7) | 4.8 (1.2, 6.1) | 0.7 |
| **Extramuscular disease activity** |  |  |  |
| Rash | 11 (52%) | 5 (56%) | >0.9 |
| Raynaud phenomenon | 3 (16%) | 2 (25%) | 0.6 |
| Arthritis | 6 (30%) | 4 (44%) | 0.7 |
| Interstitial lung disease | 7 (33%) | 3 (33%) | >0.9 |
| Dysphagia | 13 (68%) | 4 (50%) | 0.4 |
| **CV risk factors** |  |  |  |
| Dyspnea | 12 (57%) | 2 (22%) | 0.12 |
| Angina | 3 (16%) | 1 (13%) | >0.9 |
| Palpitations | 8 (42%) | 1 (13%) | 0.2 |
| Syncope | 1 (5.6%) | 1 (14%) | 0.5 |
| Blood pressure (systolic) | 136 (130, 143) | 129 (127, 139) | 0.3 |
| Blood pressure (diastolic) | 86 (77, 92) | 78 (76, 83) | 0.12 |
| BMI | 27 (25, 33) | 30 (26, 39) | 0.3 |
| Hypertension | 11 (52%) | 4 (44%) | >0.9 |
| Diabetes | 3 (14%) | 0 (0%) | 0.5 |
| Hypercholesterolemia | 16 (76%) | 3 (33%) | **0.042** |
| HbA1c | 38.0 (33.0, 38.5) | 34.0 (32.0, 37.0) | 0.4 |
| **Smoking status** |  |  | 0.8 |
| Never | 11 (52%) | 5 (56%) |  |
| Former | 9 (43%) | 3 (33%) |  |
| Current | 1 (4.8%) | 1 (11%) |  |
| TnI | 1.00 (1.00, 1.00) | 1.00 (1.00, 1.00) | 0.5 |
| TnI, > 5 ng/L | 1 (5.6%) | 0 (0%) | >0.9 |
| TnT | 14 (7, 24) | 10 (7, 13) | 0.3 |
| TnT, >14 ng/L | 9 (47%) | 1 (11%) | 0.10 |
| **ECG** |  |  |  |
| **Sinus rhythm** |  |  | >0.9 |
| 0 | 0 (0%) | 0 (0%) |  |
| 1 | 20 (100%) | 9 (100%) |  |
| Heart Rate | 73 (60, 82) | 60 (58, 84) | 0.5 |
| P-wave-duration | 80 (75, 93) | 80 (80, 85) | >0.9 |
| PQ-duration | 154 (137, 173) | 142 (136, 158) | 0.4 |
| QT-duration | 395 (379, 419) | 432 (348, 438) | 0.7 |
| QTc (Fredericia) | 421 (410, 430) | 410 (396, 427) | 0.4 |
| QTc (Bassets) | 432 (407, 440) | 417 (410, 428) | 0.3 |
| QRS-duration | 93 (83, 99) | 90 (88, 98) | 0.7 |
| QRS-duration, >120ms | 0 (0%) | 0 (0%) | >0.9 |
| **CMRI** |  |  |  |
| LVEDV | 130 (105, 164) | 157 (142, 179) | 0.090 |
| LVESV | 45 (35, 59) | 58 (41, 59) | 0.5 |
| LVEF | 65.0 (62.0, 67.0) | 67.0 (64.0, 71.0) | 0.4 |
| RVEDV | 136 (115, 171) | 162 (154, 186) | 0.15 |
| RVESV | 61 (54, 73) | 69 (68, 81) | 0.2 |
| RVEF | 58.0 (55.0, 60.0) | 58.0 (55.0, 60.0) | >0.9 |
| AoSV | 74 (61, 89) | 85 (74, 98) | 0.063 |
| PulmSV | 72 (65, 87) | 91 (83, 100) | **0.025** |
| Native T1- mapping | 995 (979, 1,012) | 1,038 (986, 1,056) | 0.12 |
| T2 - mapping | 52 (50, 55) | 60 (58, 60) | **<0.001** |
| ^1^ n (%); Mean (±SD) or Median (IQR). ^2^ Fisher’s exact test; Wilcoxon rank sum test; Wilcoxon rank sum exact test.  IIM=idiopathic inflammatory myopathy; IBM = inclusion body myositis; CV= cardiovascular; LVEDV=Left ventricle end-diastolic volumne volumenevolume; LVESV=Left ventricle end-systolic volume; LVEF=Left ventricle ejection fraction; RVEDV= Right ventricle end-diastolic volume; RVESV=Right ventricle end-systolic volume; RVEF=Right ventricle ejection fraction; AoSV= Aortic stroke volume; PulmSV= Pulmonary stroke volume. | | | |
